# The Chemical Structure and Pharmacological Activity of Sesquiterpenoids in *Dendrobium* Sw.

**DOI:** 10.3390/molecules29245851

**Published:** 2024-12-11

**Authors:** Jiaying Li, Chunxue Gao, Zaishuang He, Ya Huang, Daopeng Tan, Lin Qin, Di Wu, Jiajia Wu, Ya Wang, Yuqi He, Xingdong Wu, Yanliu Lu

**Affiliations:** 1 Key Lab of the Basic Pharmacology of The Ministry of Education, Zunyi Medical University, 6 West Xue-Fu Road, Zunyi 563000, China; jiaying.kea@foxmail.com (J.L.); zaishuang_he@foxmail.com (Z.H.); 2 Guizhou Engineering Research Center of Industrial Key-Technology for Dendrobium Nobile, Zunyi Medical University, 6 West Xue-Fu Road, Zunyi 563000, China; gaocx218@163.com (C.G.); tandp@zmu.edu.cn (D.T.); qinlin1115@163.com (L.Q.); wd_32677@126.com (D.W.); wangyaqn@foxmail.com (Y.W.); yqhe.pharm@foxmail.com (Y.H.); 3 Jinsha County Chinese Medicine Hospital, Bijie 550016, China; hy1059182353@163.com; 4 Shanghai Key Laboratory for Molecular Engineering of Chiral Drugs, School of Pharmacy, Shanghai Jiao Tong University, Shanghai 200240, China; wujiajia_1160@126.com

**Keywords:** *Dendrobium* Sw., sesquiterpenoids, structure classification, pharmacological, *Dendrobium nobile*, dendrobine

## Abstract

*Dendrobium* is one of the most important orchids with high medicinal value. The diverse pharmacological activities of *Dendrobium* are attributed to its rich content of secondary metabolites. Due to the high variety and content of sesquiterpenoids in *Dendrobium*, more studies on their pharmacological activities have been reported. More than 100 sesquiterpenoids have been isolated from the roots and stems of *Dendrobium*, and these compounds have been shown to play important roles in a variety of diseases. However, there is a lack of systematic summarization of the chemical structures and pharmacological activities of sesquiterpenoids in *Dendrobium* Sw. Here, we reviewed the chemical structures of the sesquiterpenoids in *Dendrobium* plants, classified them according to the skeleton types, and summarized their pharmacological activities and mechanisms of action, with a view to laying a foundation for future studies on their pharmacological activity mechanisms and rational clinical applications.

## 1. Introduction

*Dendrobium* Sw. is one of the most widely represented genera of Orchidaceous family. According to the “Plants of the World Online|Kew Science” database, *Dendrobium* Sw. comprises 1602 species, with the genus primarily cultivated in China, India, the Amanda Islands, Myanmar, Asia, North America, Oceania, and other tropical and subtropical regions. There are more than 80 species of *Dendrobium* in China, including *Dendrobium nobile* Lindl. (*D. nobile*), *Dendrobium candidum* (*D. candidum*), *Dendrobium huoshanense* (*D. huoshanense*), and others [1].

*Dendrobium* is used for the treatment of various diseases, medicinal diets, and horticultural ornamentals purposes, and it has a history of use in several countries such as China, Australia, India, and Malaysia. Approximately 30 species of *Dendrobium* are commonly employed, including *D. nobile*, *D. candidum*, *D. polyanthum*, *D. nobile*, *D. candidum*, *D. polyanthum*, etc. Most notably, China is the first country to use *Dendrobium* as herbal medicine, with its medicinal applications traceable to the Qin and Han dynasties [2]. In the “Shennong’s Classic of the Materia Medica”, it is recorded that *Dendrobium* is a top-ranking traditional Chinese medicine that strengthens the “yin” and nourishes the five viscera. It is also named as “the first of the nine immortal medicines” among the many precious Chinese herbs [3]. As evidenced in the extant literature, over 30 species of *Dendrobium* have been employed in the treatment of various diseases in China [4]. Phytochemical studies have demonstrated that *Dendrobium* possesses a multitude of pharmacologically active compounds, including alkaloids [5,6,7,8,9,10,11,12,13,14,15], sesquiterpenoids [16,17,18,19,20,21,22,23,24,25], flavonoids [10,19,26], phenanthrenes [20,27,28,29], and polysaccharides [30,31,32]. In particular, sesquiterpenoids in *Dendrobium* Sw. (*DSS*), which is abundant and structurally diverse and exhibits potent pharmacological activities, have received increasing attention from researchers [20,33,34].

Sesquiterpenoids are naturally occurring terpenoids comprising 15 carbon atoms in the molecule, containing three isoprene units, and exhibiting a range of skeletal structures, including chains and rings. The components of this class are widely distributed, and the majority of plants continuously synthesize a variety of sesquiterpenoids during their growth and development stages, including *Dendrobium* Sw. In recent years, research has demonstrated that sesquiterpenoids are the most prevalent and abundant class of compounds in the genus *Dendrobium*. These compounds have been shown to play a crucial role in neuroprotection [35], immunomodulation [36], the treatment of diabetes [37], anti-tumor activity [38], and other biological processes. Until now, a total of 12 species of *Dendrobium* have been identified as containing sesquiterpenoids. However, a dearth of systematic syntheses exists regarding the applications of *DSS* in research. Given the extensive range and diversity of *DSS* observed in *Dendrobium*, an exhaustive review of this subject is of paramount importance. Consequently, this review aims to elucidate the current understanding of *DSS*, encompassing its structural and typological variations, as well as its pharmacological effects. The review will serve as a foundation for future investigations into its pharmacological activities and rational clinical applications.

## 2. Chemical Structure of *DSS*

It has been reported that more than 100 sesquiterpenoids have been isolated from the roots and stems of *Dendrobium*. These sesquiterpenoids can be categorized into the following types: picrotoxane-type, allo-aromadendrane-type, copacamphane-type, cyclocopacamphane-type, cadinene-type, emmotin-type, muurolene-type, axane-type, and ylangene-derived types. Among them, the picrotoxane-type is the most abundant sesquiterpenoid in *Dendrobium*, which contains more than 70 compounds. This review summarizes the sesquiterpenoid-type constituents isolated from different types of *Dendrobium* Sw. according to the skeleton type of the compounds.

### 2.1. Picrotoxane-Type Sesquiterpenoids

Picrotoxane-type sesquiterpenoids are the precursor substance of other sesquiterpenoids in *Dendrobium* Sw. To date, 75 compounds of picrotoxane-type sesquiterpenoids have been isolated from 12 species of *Dendrobium* Sw., including *D. nobile*, *Dendrobium tasselii* (*D. tasselii*), and *Dendrobium tympani* (*D. tympani*). This type of sesquiterpenoids covers over 50% of the reported sesquiterpenoids. Additionally, the available literature reveals that picrotoxane-type sesquiterpenoids in *Dendrobium* Sw. are the primary precursors in the biosynthetic pathway of most active alkaloids. It is of significant importance to summarize the class of alkaloid sesquiterpenoids in *Dendrobium* Sw., as they represent the primary active components in the majority of medicinal *Dendrobiums*. The picrotoxane-type sesquiterpene substituents can be subdivided into two types according to their characteristics: picrotoxane-type alkaloid sesquiterpenoids and picrotoxane-type nonalkaloid sesquiterpenoids. This section provides a summary of the picrotoxane-type sesquiterpene substituents according to their characteristics.

#### 2.1.1. Picrotoxane-Type Alkaloid Sesquiterpenoids

**Figure 1:**
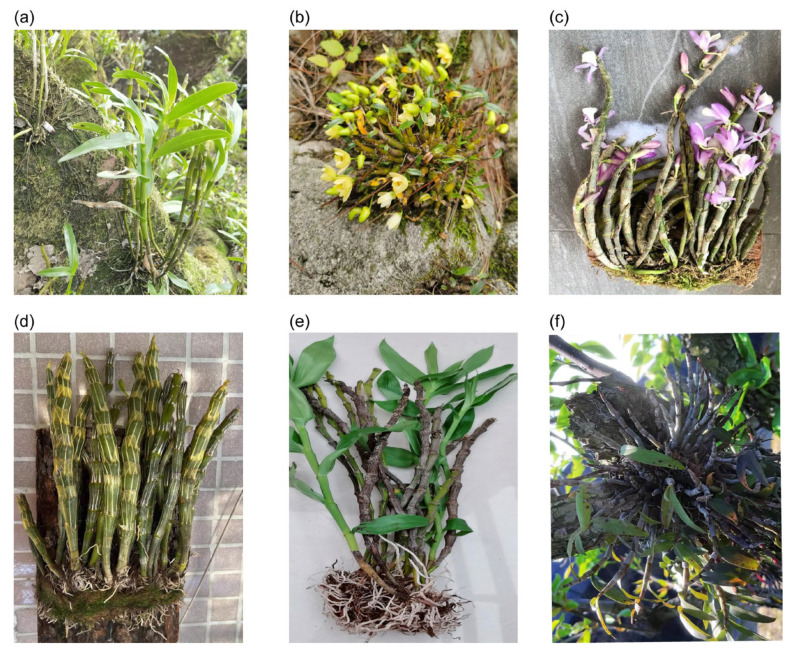
Pictures of 6 species of *Dendrobium*. (**a**) *D. nobile*; (**b**) *D. huoshanense*; (**c**) *D. polyanthum*; (**d**) *D. crepidatum*; (**e**) *D. wardianum*; (**f**) *D. candidum*.

**Figure 2:**
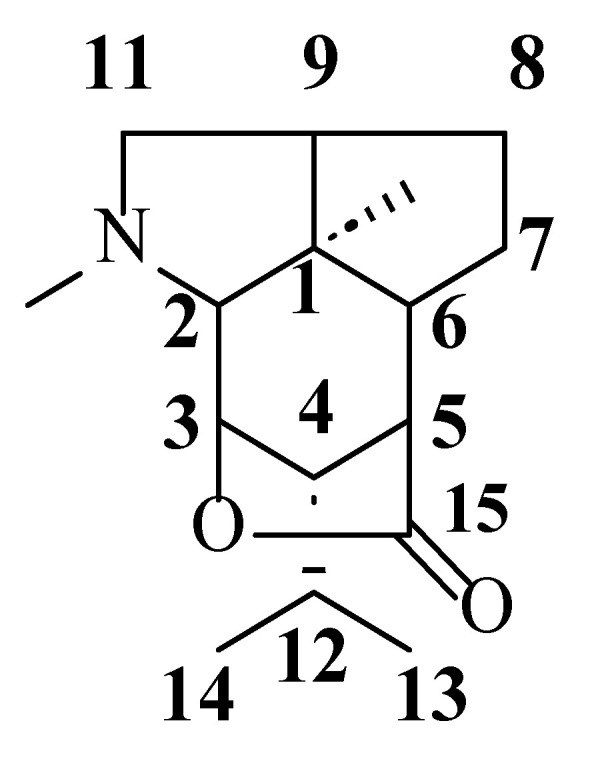
Structural skeleton of picrotoxane-type alkaloidal sesquiterpenoids. The numbers in the structure indicate the number of carbon atoms in the molecule.

**Figure 3:**
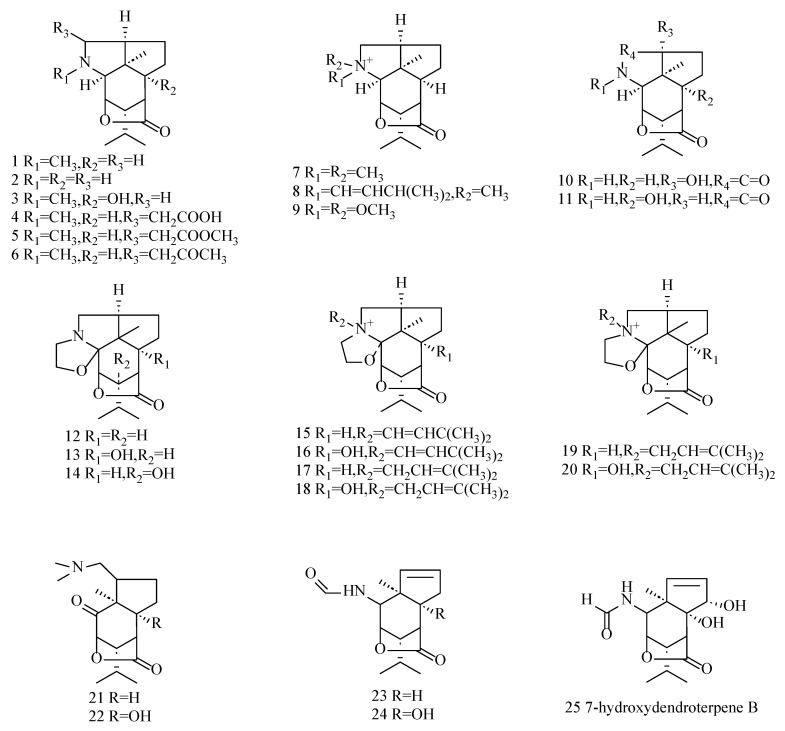
Structures of picrotoxane-type alkaloid sesquiterpenoids isolated from *Dendrobium*.

Picrotoxane-type alkaloidal sesquiterpenoids, also known as dendrobine-type alkaloidal sesquiterpenoids, are represented by the endemic constituent dendrobine of *D. nobile* [9]. The skeleton of these sesquiterpenoids is the interaction of the C_15_ position (carboxyl group) with the C_3_ position (hydroxyl group), which condenses to form a five-membered lactone ring. Figure 1 shows the structural parentheses of picrotoxane-type alkaloid sesquiterpenoids. This class of sesquiterpenoids exhibits considerable structural diversity, with variations in the skeleton primarily concentrated around the N atom and N heterocycle. The components of this class are distributed more widely in *D. nobile* [39], *D. findlayanum*, *D.* signatum [8], and *D. friedericksianum* [5]. Additionally, *D. nobile*, *D. signatum*, and *D. friedericksianum* have also been used as a quality marker [9]. The structural information is shown in Figure 2, and the available information on this class of constituents is shown in Table 1 and Figure 3.

#### 2.1.2. Picrotoxane-Type Non-Alkaloidal Sesquiterpenoids

**Figure 4:**
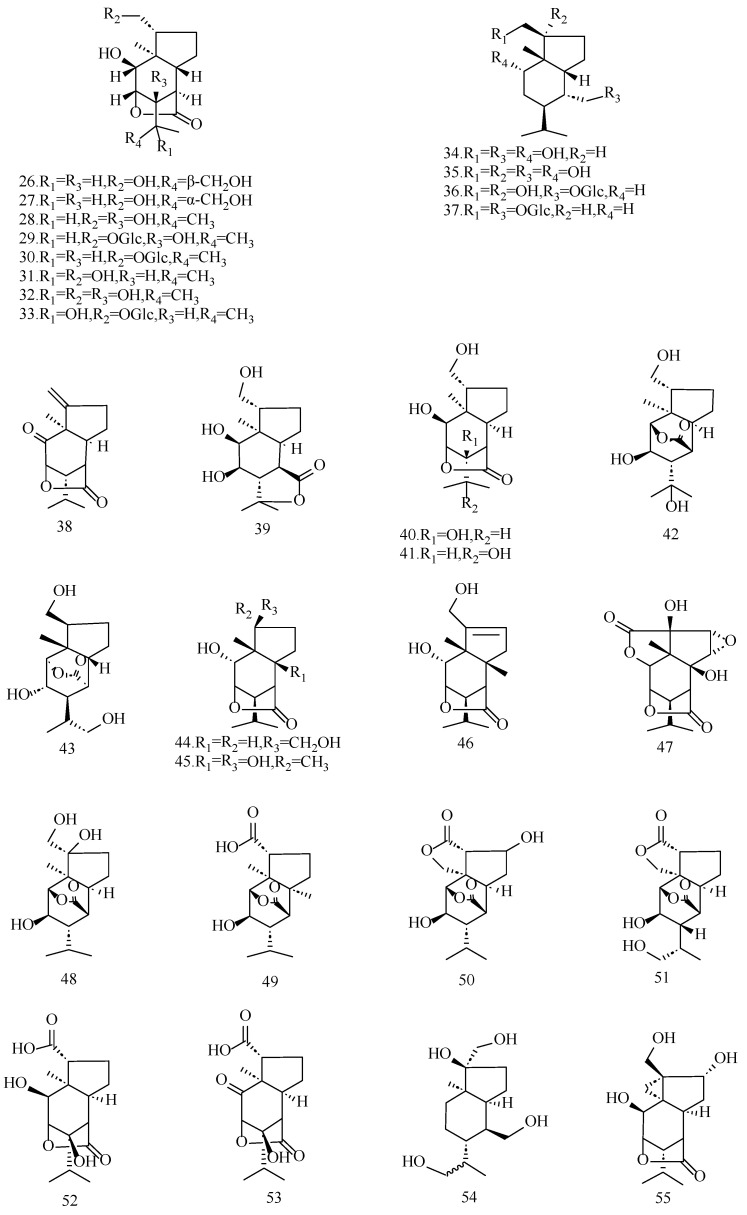

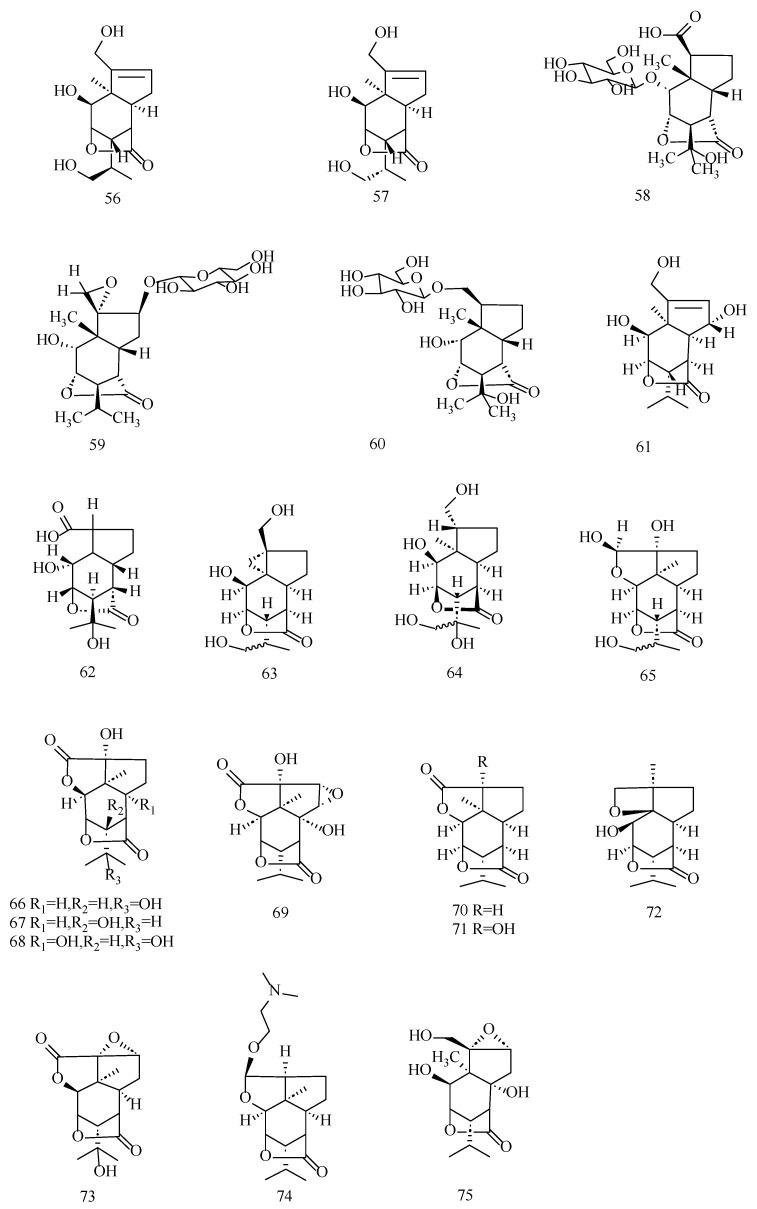
Structures of picrotoxane-type non-alkaloid sesquiterpenoids isolated from *Dendrobium*.

Picrotoxane non-alkaloidal sesquiterpenoids are characterized by a higher degree of oxygen substitution, predominantly occurring at the C4, C9, C11, and C12 positions, which often leads to the formation of polyhydroxy structures. These type of components are reported in *D. nobile* [44], *D. candidum* [22], *D.moniliforme* [45], and *D. wardianum* [18,29], especially in *D. nobile* and *D. wardianum*. Picrotoxane non-alkaloidal sesquiterpenoids are similar in skeleton to their alkaloidal sesquiterpenoids counterparts, and they are mainly characterized by having a high number of chiral carbons and the lack of nitrogen atoms in the molecule. Therefore, the absolute configuration of the components of this class is more difficult to determine. The chemical composition information of the picrotoxane class of non-alkaloidal sesquiterpenoids is shown in Table 2, and the specific structures are shown in Figure 4.

### 2.2. Allo-Aromadendrane-Type Sesquiterpenoids

**Figure 5:**
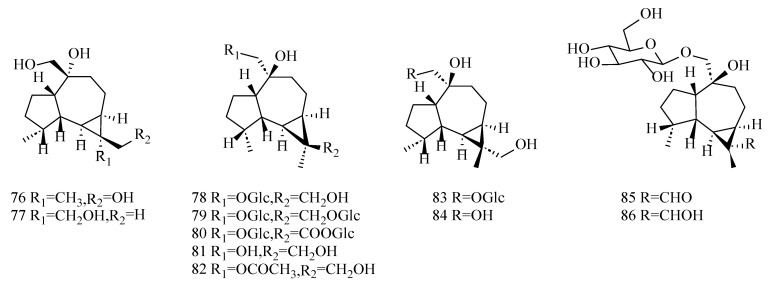
Structures of allo-aromadendrane-type sesquiterpenoids isolated from *Dendrobium*.

Allo-aromadendrane-type sesquiterpenoid structures are characterized by the presence of a cyclopropane structure. These compounds usually undergo hydroxyl substitution at the C10, C11, and C13 positions, with their glucose often binding at the C13 and C15 positions, resulting in the formation of sesquiterpene glycosides. By analyzing this class of constituents, it can be found that this class of constituents mainly exists in *D. nobile* and is rarely reported in *D. findlayanum* and *D. moniliforme*. The compositional information table and structure is shown in Table 3 and Figure 5.

### 2.3. Copacamphane- and Cyclocopacamphane-Type Sesquiterpenoids

**Figure 6:**
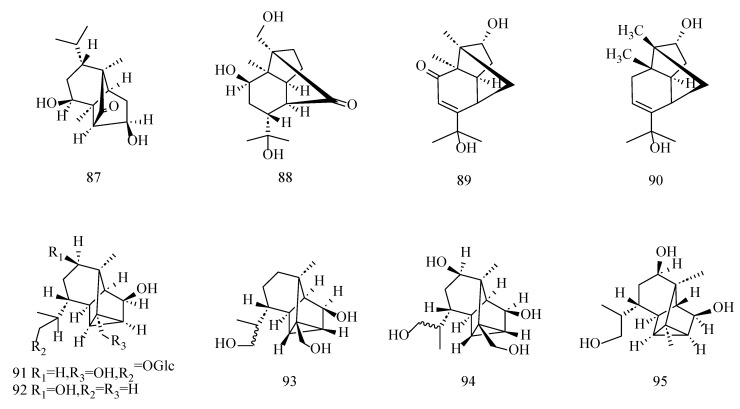
Structures of copacamphane- and cyclocopacamphane-type sesquiterpenoids isolated from *Dendrobium*.

The structural similarity between cyclocopacamphane-type sesquiterpenoids and copacamphane-type sesquiterpenoids in *Dendrobium* Sw. is extremely high. The major difference between them is that cyclocopacamphane-type sesquiterpenoids can form ternary rings, mainly at the C8 and C9 positions. In addition, hydroxyl substitution can also occur in this group of components, mainly at the C4 and C8 positions. Detailed information of cyclocopacamphane-type and copacamphane-type sesquiterpenoids is shown in Table 4, and their structures can be seen in Figure 6.

### 2.4. Cadinene-Type Sesquiterpenoids

**Figure 7:**
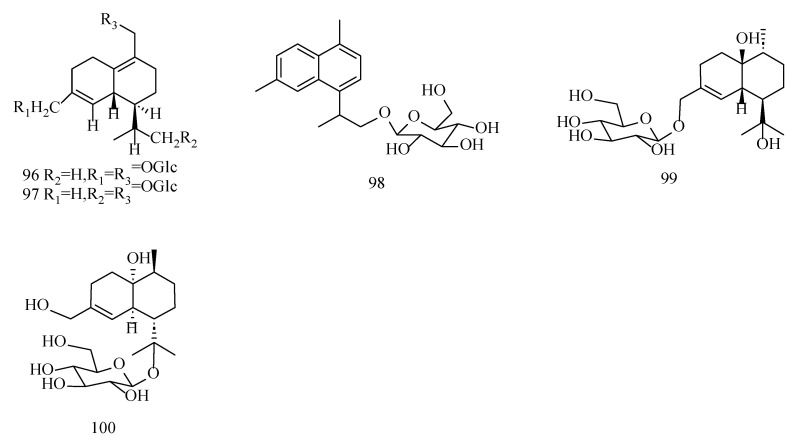
Structures of cadinene-type sesquiterpenoids isolated from *Dendrobium*.

Cadinene-type sesquiterpenoids have been isolated from both *D. nobile* and *D. findlayanum*, with five components currently reported. These compounds can usually be combined with glucose moieties to form glycosidic components. The compounds’ information is shown in Table 5 and the structures in Figure 7.

### 2.5. Other Types of Sesquiterpenoids 

**Figure 8:**
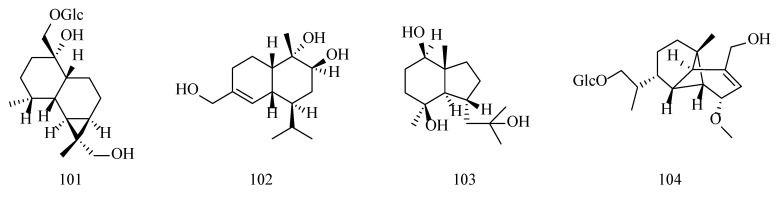
Structures of emmotin-, murolene-, axane-, and ylangene-derived-type sesquiterpenoids isolated from *Dendrobium*.

In addition, *Dendrobium* Sw. also contains other types of sesquiterpenoids, including emmotin-type sesquiterpenoids, muurolene-type sesquiterpenoids, axane-type sesquiterpenoids, and ylangene-derived sesquiterpenoids. Their compositional information and structures are shown in Table 6 and Figure 8.

## 3. Pharmacological Activity

*DSS* exhibits diverse pharmacological activities, especially sesquiterpene alkaloids and sesquiterpene glycosides. For example, *D. nobile*, whose pharmacological activities have been studied by focusing on total alkaloids extracted from *D. nobile* (DNLA) and its representative compound dendrobine, has shown significant effects in blood glucose regulation, neuroprotection, anti-tumor activity, etc. In addition, the pharmacological effects on immunoinflammatory modulation, antiviral, and other aspects of other classes of components in *Dendrobium* have also been reported, such as sesquiterpene glycoside components. The aim of this section is to summarize the pharmacological activity and mechanism of action of *DSS*, and to provide research progress for its further product development and clinical application.

### 3.1. Treatment of Diabetes

DNLA has significant therapeutic effects on diabetes. Its regulation of blood glucose is summarized in three aspects: increasing insulin sensitivity, glycogen synthesis, and decreasing lipid synthesis. DNLA mainly regulates genes and pathways related to glucose metabolism, insulin action, and lipid metabolism to promote the balance of glucose metabolism in the body.

In improving insulin sensitivity, Shihunine, a water-soluble alkaloid in *D. meridianum*, significantly reduces body weight, fasting blood glucose, and lipid levels in db/db mice. It also significantly improves the oral glucose tolerance test (OGTT) and serum insulin levels. The underlying mechanism may involve the agonistic adenosine monophosphate (AMP)-activated protein kinase phosphorylation (p-AMPK). It also involves increased expression levels of peroxisome proliferator-activated receptor alpha (PPARα) and glucose transporter protein 4 (GLUT4) in liver and adipose tissue [60]. In regulating glycogen synthesis, DNLA increases the expression of PGC1α in both the mRNA and protein levels. Moreover, DNLA enhances the expression of glucose metabolism genes such as glucose transporter protein 2 (GLUT2) and Forkhead box transcription factor O1 (FoxO1) [35]. In addition, *D. nobile* regulated blood glucose levels in HFD and STZ-induced diabetic rats. Its glucose-lowering mechanism may be associated with the activation of the PI3K/AKT pathway and insulin signaling pathway, which regulate hepatic glycogen synthesis and gluconeogenesis [59]. In the regulation of lipid synthesis, DNLA up-regulates fatty acid β-oxidation genes, including acyl-CoA oxidase 1(Acox1) and Carnitine Palmitoyl-Transferase1A (Cpt1a). Concurrently, a decrease in the lipid synthesis regulator Sterol Regulatory Element Binding Protein-1 (Srebp1) and an increase in the lipolysis gene Adipose Triglyceride Lipase (ATGL) were observed [35].

Furthermore, DNLA also reduced the amount of Th17 in gestational diabetic mice, which can effectively alleviate gestational diabetes in mice and may serve as a potential therapeutic candidate for gestational diabetic patients [61]. The alcoholic extract of *D. bulbosum* ameliorated hyperglycemia and diabetic complications in STZ-induced diabetic rats by inhibiting the expression of pro-angiogenic factors, including VEGF/VEGFR2, MMP 2/9, PDGF A/B, bFGF, and insulin-like growth factor 1 (IGF-1) [27].

**Figure 9:**
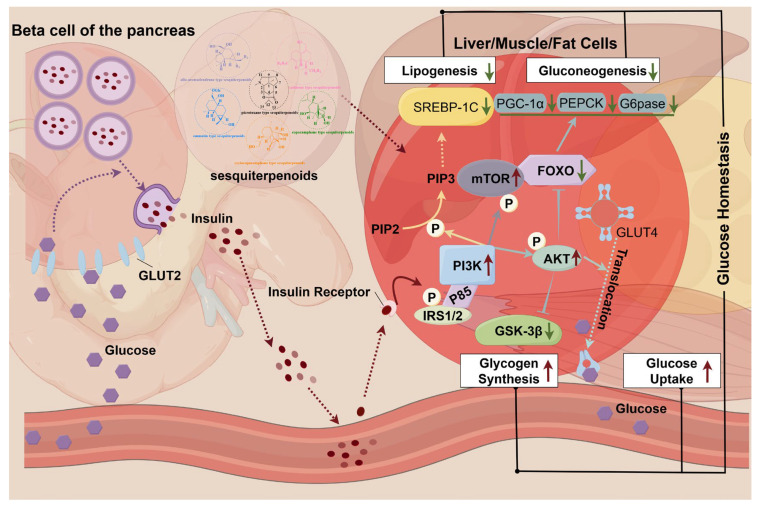
Antidiabetic mechanism of *DSS*. Solid arrows are direct effects; dashed arrows are indirect effects. P is phosphorylation, red upward arrows are up-regulated; green downward arrows are down-regulated. Glucose transporter 2/4 (GLUT2/4), insulin receptor substrate (IRS1/2), phosphatidylinositol 3-kinase (PI3K), mechanistic target of rapamycin(mTOR), glycogen synthase kinase-3 beta (GSK-3β), protein kinase B (AKT), Forkhead box O (FOXO), Peroxisome Proliferator-Activated Receptor Gamma Coactivator-1 Alpha (PGC-1α), phosphoenolpyruvate carboxykinase (PEPCK), glucose-6-phosphatase (G6pase), phosphatidylinositol-4,5-bisphosphate (PIP2), phosphatidylinositol-3,4,5-trisphosphate (PIP3).

These findings suggest that the sesquiterpenoids in *Dendrobium* have therapeutic potential for the treatment of diabetes mellitus and may be beneficial in lowering blood glucose, improving insulin resistance, and alleviating diabetes-related complications. Their mechanism of action is shown in Table 7 and Figure 9.

### 3.2. Neuroprotection

*DSS* protects neurons from neurodegenerative diseases, including Alzheimer’s disease (AD), Parkinson’s disease (PD), and Huntington’s disease. DNLA is the primary sesquiterpene constituent of *Dendrobium* and has been reported to exert neuroprotective effects in modern pharmacological studies. And its neuroprotective mechanism can be summarized in two aspects: the reduction of protein overaccumulation and the inhibition of inflammatory cytokine production.

DNLA significantly improves streptozotocin-induced damage and loss of hippocampal neurons in rat, activates GSK-3β, and prevents the hyperphosphorylation of Tau proteins [63]. In addition, reports in the literature indicate that DNLA can reduce the levels of BACEl and Aβ in hippocampal neurons [64]. DNLA may protect neuronal synaptic integrity by upregulating the synaptophysin and postsynaptic dense area protein-95 [65]. DNLA improves cognitive dysfunction and neuronal damage in aged SAMP8 mice while reducing abnormal protein accumulation and gene expression [66].

It has been demonstrated that *DSS* inhibits oxidative stress and neuroinflammation while reducing neurotoxicity [67,68]. Additionally, *DSS* inhibits oxidative stress and neuroinflammation, reduces neurotoxicity [60], and mitigates brain damage [63,67,69] and neuropathy [63,65,70]. DNLA has been shown to prevent oxidative stress-induced diseases in different neurological disorders, providing an experimental basis for its potential application [67]. DNLA can prevent CIR injury by inhibiting neuronal death, thus providing new therapeutic insights into CIR injury [71].

**Figure 10:**
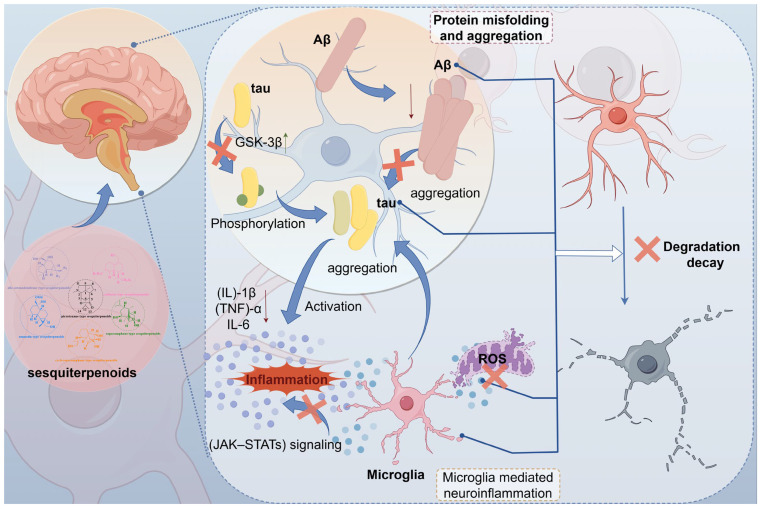
Mechanism of neuroprotective action of *DSS*. Green upward arrows are for upward adjustments, and red downward arrows are for downward adjustments. Microtubule-associated protein tau (tau); microtubule-associated protein tau. Reactive Oxygen Species (ROS), amyloid-β (Aβ), glycogen synthase kinase-3 beta (GSK-3β), interleukin-1β (IL-1β), Tumor Necrosis Factor-α (TNF-α), interleukin-6 (IL6).

Furthermore, newly isolated sesquiterpenoids from *D. hercoglossum* have demonstrated significant neuroprotective properties [16]. In addition, DNLA is distributed in the brain tissue of C57BL/6J mice, indicating that it can cross the blood–brain barrier, thus providing an experimental basis for the neuroprotective effects of sesquiterpenoids in *Dendrobium* [72]. Their mechanism of action is shown in Table 8 and Figure 10.

### 3.3. Anti-Tumor

Alkaloid sesquiterpenes from *Dendrobium* Sw. possess a novel structure and rich pharmacological effects, particularly in anti-tumor performance, highlighting their potential for new drug development. The representative alkaloid in *Dendrobium* genus is dendrobine, which is based on the picrotoxane skeleton and has been found to exert inhibitory effects on various tumors [17,80,81,82]. The primary anti-tumor mechanism of dendrobine involves inhibiting the activation of proto-oncogenes and increasing the expression of oncogenes, thus suppressing the abnormal proliferation and differentiation of cells. The related mechanisms are as follows.

Dendrobine, by targeting JNK stress signaling, enhances cisplatin toxicity of in vivo during chemotherapy for non-small cell lung cancer cells [80]. A picrotoxane-type sesquiterpene (dendrowardin E, amotin, aduncin) from *D. findlayanum* exhibits significant effects in promoting D-galactose-induced proliferation of Human Lens Epithelium Cells (HLECs) [53]. A newly isolated ylangene-type sesquiterpene glycoside from *D. findlayanum* exhibits toxic activity against human cancer cell lines SMMC-7721, A-549 and MCF-7 [17]. The ethanolic extract of *D. fascicularis* induced apoptosis by upregulating p53 in HeLa cells and inhibited tumor progression in mice [81]. Interestingly, the anti-tumor-related target gene p53 associated with dendrobine and its classical target gene p21 are closely linked to the development of aging and aging-related diseases [83]. The relevant literature indicates that dendrobine exhibits anti-aging activity. However, the mechanisms of action require further research.

**Figure 11:**
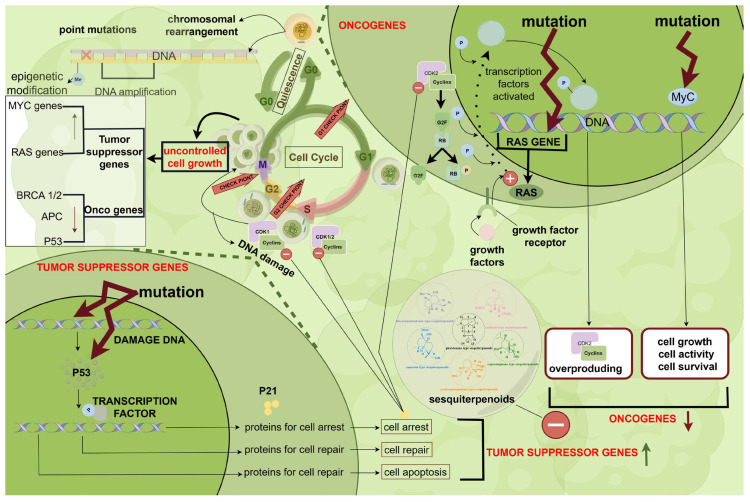
Anti-tumor mechanism of action of *DSS*. Green upward arrows are for upward adjustments; red downward arrows are for downward adjustments. The white horizontal line in a red circle represent inhibition, and the white cross in a red circle represents activation. P is phosphorylation. Cyclin-Dependent Kinase 1/2 (CDK1/2).

In conclusion, *DSS* has anti-tumor activity, inhibiting the growth and metastasis of tumor cells while improving the efficacy of chemotherapeutic drugs. In addition, this class of ingredients has anti-aging effects. However, studies on the anti-tumor effects of *DSS* are largely limited to in vitro experiments. Further validation of its effects and elucidation of its mechanisms are needed. The results of this study provide a scientific basis for the clinical application of *DSS* and serve as a reference for the development of new anti-tumor and anti-aging drugs. Their mechanisms of action are shown in Table 9 and Figure 11.

### 3.4. Immuno and Anti-Inflammatory Activity

*DSS* was found to have significant immunomodulatory effects. The compounds dendroside A, dendroside D, dendroside F, dendroside G, and dendronobiloside A have promotional effects in the proliferation of T and B lymphocytes [46,47]. In addition, dendronobiloside B showed inhibitory efficacy in terms of immune functions [51] and also showed inhibitory effects on immune function. Dendroside E also exhibits immunologic activity [47].

Dendronbiline D, a compound isolated from *D. nobile*, exhibited significant anti-inflammatory activity [16]. Three new sesquiterpenoids isolated from *D. nobile*, namely dendronbiline A, B, and C, exhibited significant anti-inflammatory activity against LPS-treated RAW 264.7 cells. Three new sesquiterpenoids isolated from *D. reuteri*, namely *Dendrobium* terpenoids A, B, and C, exhibited inhibitory activity against NO production in LPS-treated RAW 264.7 cells [17]. Dendrobine alleviates liver injury in metabolic dysfunction-associated steatotic liver disease (MASLD). Its mechanism may involve regulating inflammatory and immune responses and affect lipid metabolism by down-regulating inflammatory mediators (e.g., TNF, IL6, IL1B), inhibiting AKT1, suppressing transcriptional signaling, and activating factor 3 (STAT3) [84]. The *D. chrysogenum* sesquiterpene-RTK axis upregulates ELF4 expression through activation of the ERK1/2 signaling pathway, and ELF4 up-regulation inhibits inflammatory responses through trans-activation of S100A9 [24].

In conclusion, *DSS* exhibits notable immunomodulatory and anti-inflammatory activities, but the studies on these compounds have primarily been conducted in vitro, and the specific pharmacological activities and mechanisms require further investigation. Their mechanisms of action are shown in Table 10.

### 3.5. Other Pharmacological Activities

Studies have shown that the alkaloidal sesquiterpenoid dendrobine possesses viral inhibitory activity and antiviral activity. The mechanism study showed that dendrobardine can inhibit the early steps of viral replication cycle [85]. In addition, dendrowardin E significantly promoted the proliferation of HLECs induced by D-galactose [46].

## 4. Discussion

The structural classification, pharmacological effects, and mechanism of action of *DSS* are summarized by a review. Over 100 sesquiterpenoids have been isolated from the roots and stems of 12 *Dendrobium* species, which can be categorized into 9 types based on their backbone structure. The picrotoxane type is the most abundant and has been reported to possess a greater number of pharmacological activities. These activities include the regulation of blood glucose, neuroprotection, immunomodulation, anti-tumor activity, and anti-inflammatory effects. Dendrobine, a representative sesquiterpenoid compound, was employed as a quality marker for assessing and identifying *D. nobile*. Clearly, relying on a single indicator is insufficient to evaluate its quality. This indicates that other sesquiterpenes could serve as potential quality markers for *DSS*, although they are not yet documented in the Chinese Pharmacopoeia. Therefore, future studies on *DSS* could focus on species with documented usage that have been less extensively studied.

Despite the diverse pharmacological activities of *DSS*, there are still few reports on its clinical applications and mechanistic studies. Therefore, the specific mechanism of action of *DSS* remains unclear. Furthermore, in some cases, pharmacological activity may only be observed at high doses of *Dendrobium* extracts in clinical applications. Consequently, detailed and large-scale clinical studies are necessary to provide sufficient evidence to ensure drug efficacy and patient safety. Due to their commercial value, most studies have focused on the dominant species (*D. nobile.*, *D. candidum*, *D. huoshanense*), resulting in the neglect of sesquiterpenoid constituents in other *Dendrobium* species. In addition, a general lack of systematic studies on the pharmacokinetic aspects of *DSS* exists, which should be studied in greater depth to improve their bioavailability.

For others, sesquiterpenoids with immunomodulatory effects are primarily classified as picrotoxanes, a non-alkaloidal sesquiterpenoid found in *Dendrobium*. These sesquiterpenoids are predominantly sesquiterpene glycosides, with most research focusing on their isolation and in vitro studies. However, their pharmacological activities remain largely unexplored, offering a valuable avenue for further investigation and potential advancement in this field. The role of other types of sesquiterpenoids in immunity needs to be further explored. In addition, *DSS* shows anti-inflammatory activity, but there are few studies on it, so the research on the immunomodulatory and anti-inflammatory activities of this genus is very promising.

## 5. Conclusions and Future Directions

*DSS* has been extracted and isolated from various *Dendrobium* species. However, most studies are focused on a few species of *Dendrobium* with high commercial value. These studies are primarily focused on a small number of compounds such as alkaloidal sesquiterpenes and sesquiterpene glycosides.

Although there is an increasing number of research reports on *DSS*, which has shown significant pharmacological activities in the treatment of diabetes and its complications, neuroprotection, anti-tumor effect, and immuno-inflammatory regulation, most of the current studies remain superficial. These studies primarily focus on observing the changes induced by *DSS* administration, but they fail to clearly explain its underlying mechanisms of action.

The potent pharmacological activity of *DSS* may be attributed to its structural diversity. These compounds can be classified into nine types according to the skeleton, and the substituent types of these compounds are also extremely diverse. Although the pharmacological effects and mechanisms of action have been explored, they remain insufficiently detailed. Additionally, further refinement is needed in the areas of pharmacotoxicology and pharmacokinetics. However, continued studies on the structural identification and pharmacological activity of *DSS* may be fruitful in the clinical application and novel drug development of *Dendrobium* Sw.

## Figures and Tables

**Table 1:** Information table of picrotoxane-type alkaloid sesquiterpenoids isolated from *Dendrobium*.

No.	Compounds	Formula	Sources	Refs.
1	Dendrobine	C_16_H_25_NO_2_	*D. nobile*, *D. crepidatum*	[22,31,40,41]
2	Mubironine B	C_15_H_23_NO_2_	*D. nobile*	[41]
3	Dendramine	C_16_H_25_NO_3_	*D. nobile*	[13,41]
4	(-)-(1*R*,2*S*,3*R*,4*S*,5*R*,6*S*,9*S*,11*R*)-11-Carboxy-methyl-dendrobine	C_18_H_27_NO_4_	*D. nobile*	[40]
5	Dendrine	C_19_H_29_NO_4_	*D. nobile*	[40]
6	Dendronobiline A	C_19_H_29_NO_3_	*D. nobile*	[15]
7	N-methyl-dendrobinium	C_17_H_28_NO_2_^+^	*D. nobile*	[41]
8	N-isopentenyl-dendrobinium	C_21_H_34_NO_2_^+^	*D. nobile*	[41]
9	Dendrobine N-oxide	C_16_H2_5_NO_3_	*D. nobile*	[7]
10	3-Hydroxy-2-oxodendrobine	C_16_H_23_NO_4_	*D. nobile*	[42]
11	Oxodendramine	C_16_H_23_NO_4_	*D. nobile*	[42]
12	Dendroxine	C_17_H_25_NO_3_	*D. nobile*	[12,14]
13	6-Hydroxy-dendroxine	C_17_H_25_NO_4_	*D. nobile*	[14]
14	4-Hydroxy-dendroxine	C_17_H_25_NO_4_	*D. nobile*	[14]
15	N-Isopentenyl-dendroxinium	C_22_H_34_NO_3_^+^	*D. nobile*	[41]
16	N-Isopentenyl-6-hydroxydendroxinium	C_22_H_34_NO_4_^+^	*D.* *nobile.*	[41]
17	N-Isopentenyl-dendroxinium chloride	C_22_H_34_NO_3_^+^	*D. nobile*	[7,41]
18	N-Isopentenyl-6-hydroxydendroxinium chloride	C_22_H_34_NO_4_^+^	*D. nobile*	[7,41]
19	N-isopentenyl-dendroxine	C_22_H_34_NO_3_^+^	*D. nobile*, *D. friedericksianum*	[43]
20	N-isopentenyl-6-hydroxy-dendroxine	C_22_H_34_NO_4_^+^	*D. nobil*, *D. friedericksianum*	[43]
21	Nobilonine	C_17_H_27_NO_3_	*D. nobile*, *D. crepidatum*	[41]
22	6-Hydroxynobiline	C_17_H_27_NO_4_	*D. nobile*	[13,40,41]
23	Dendroterpene A	C_15_H_21_NO_3_	*D. nobile*	[44]
24	Dendroterpene B	C_15_H_21_NO_4_	*D.* *nobile.*	[44]
25	7-hydroxydendroterpene B	C_15_H_21_NO_5_	*D. signatum*	[44]

**Table 2:** Information table of picrotoxane-type non-alkaloid sesquiterpenoids isolated from *Dendrobium*.

No.	Compounds	Formula	Sources	Refs.
26	Dendronobilin D	C_15_H_24_O_5_	*D. nobile*	[46]
27	Dendronobilin E	C_15_H_24_O_5_	*D. nobile*	[46]
28	Dendronobilin B	C_15_H_24_O_5_	*D. nobile*	[22,46]
29	Dendroside G (7,11-dihydroxy-5-hydroxymethyl-11-isopropyl-6-methyl-9-oxatricyclo [6.2.1.02,6] undecan-10-one-15-*O-β*-D-glucopyranoside)	C_21_H_34_O_10_	*D. nobile*	[47]
30	Dendroside F (7-hydroxy-5-hydroxymethyl-11-isopropyl-6-methyl-9-oxatricyclo [6.2.1.02,6] undecan-10-one-15-*O-β-D*-glucopyranoside)	C_21_H_34_O_9_	*D. nobile*	[47]
31	Dendrodensiflorol	C_15_H_24_O_5_	*D. nobile*	[41,48]
32	Dendronobilin L	C_15_H_24_O_6_	*D. nobile*	[46]
33	7,12-dihydroxy-5-hydroxymethyl-11-isopropyl-6-methyl-9-oxatricyclo [6.2.1.02,6] dendecane-10-one-15-*O-β*-D-glucopyranoside	C_21_H_34_O_10_	*D. nobile*	[49]
34	10,12-dihydroxypicrotoxane	C_15_H_28_O_2_	*D. nobile*	[49,50]
35	6 *α*,10,12-trihydroxypicrotoxane	C_15_H_28_O_3_	*D. nobile*	[49,50]
36	Dendronobiloside B	C_21_H_38_O_8_	*D. nobile*	[51]
37	Dendronobiloside A	C_21_H_34_O_8_	*D. nobile*	[51,52]
38	Nobilomethylene	C_15_H_20_O_3_	*D. nobile*	[14]
39	Findlayanin (1*R*,2*S*,3*R*,4*S*,5*R*,6*S*,9*R*)-2,3,11,12-tetrahydroxypicrotoxan-12(15)-lactone	C_15_H_24_O_5_	*D. nobile*, *D. findlayanum*	[22,53]
40	(+)-(1*R*,2*S*,3*S*,4*R*,5*R*,6*S*,9*R*)-2,4,11-Trihydroxypicrotoxane-3(15)-lactone	C_15_H_24_O_5_	*D. nobile*	[22]
41	(+)-(1*R*,2*S*,3*R*,4*S*,5*R*,6*S*,9*R*)-2,11,12-Trihydroxypicrotoxane-3(15)-lactone	C_15_H_24_O_5_	*D. nobile*	[22]
42	(+)-(1*R*,2*S*,3*R*,4*S*,5*R*,6*S*,9*R*)-3,11,12-Trihydroxypicrotoxane-2(15)-lactone	C_15_H_24_O_5_	*D. nobile*	[21]
43	(−)-(1*S*,2*R*,3*S*,4*R*,5*S*,6*R*,9*S*,12*R*)-3,11,13-Trihydroxypicrotoxane-2(15)-lactone	C_15_H_24_O_5_	*D. nobile*	[21]
44	Dendrobiumane B	C_15_H_24_O_4_	*D. moniliforme*	[45]
45	Dendrobiumane D	C_15_H_24_O_5_	*D. moniliforme*	[45]
46	Dendrobiumane C	C_16_H_24_O_4_	*D. moniliforme*	[45]
47	Dendrobiumane E	C_15_H_18_O_7_	*D. moniliforme*	[45]
48	Dendrowardin A ((1*S*,2*S*,3*R*,4*S*, 5*R*,6*S*,9*S*)-3,9,11-trihydroxypicrotoxane-2(15)-lactone.)	C_15_H_24_O_5_	*D. wardianum*	[18]
49	Dendrowardin B ((1R*,2S*,3R*,4S*,5R*,6S*,9R*)-9-carboxy-9-desmethyl-3-hydroxypicrotoxano-2(15)-lactone)	C_15_H_22_O_5_	*D. wardianum*	[18]
50	Dendrowardin C ((1R*,2S*,3R*,4S*,5R*,6S*,8R*,9R*)-3,8- dihydroxypicrotoxano-2(15),10(11) -dilactone)	C_15_H_20_O_6_	*D. wardianum*	[18]
51	Dendrowardin D ((1R*,2S*,3R*,4S*,5R*,6S*,9R*,12S*)-3,13-dihydroxypicr otoxano-2(15),10(11)-dilactone.)	C_15_H_20_O_6_	*D. wardianum*	[18]
52	Dendrowardin E ((1R*,2S*,3S*,4R*,5R*,6S*,9R*)-9-carboxy-9-desmethyl-2,4-dihydroxypicrotoxano-3(15)-lactone)	C_15_H_22_O_6_	*D. wardianum*	[18]
53	Dendrowardin F ((1R*,3R*,4R*,5R*,6S*,9R*)-9-carboxy-9-desmethyl-4-hydroxypicrotoxano-3(15)-lactone-2-one)	C_15_H_20_O_6_	*D. wardianum*	[18]
54	Dendrowardin I ((1R*,4S*,5S*,6S*,9S*)-9,11,13,15- tetrahydroxypicrotoxane)	C_15_H_26_O_4_	*D. wardianum*	[18]
55	Dendrowardin J ((1R*,2S*,3R*,4S*,5R*,6S*, 8R*,9S*)-9,10-cyclo-2,8,11-trihydroxypicrotoxano-3(15)-lactone)	C_15_H_22_O_5_	*D. wardianum*	[18]
56	Dendrowardin G ((1R*,2S*,3R*,4S*,5R*,6S*,12R*)-2,11,13-trihydroxypic rotoxano-8-en-3(15)-lactone)	C_15_H_22_O_5_	*D. wardianum*	[18]
57	Dendrowardin H ((1R*,2S*,3R*,4S*,5R*,6S*,12S*)-2,11,13- trihydroxypic rotoxano-8-en-3(15)-lactone)	C_15_H_22_O_5_	*D. wardianum*	[18]
58	Dendromoniliside B (2α,3α,12-trihydroxypicro- toxane-3(15α)-olid-11-oic acid 2-*O-β-D*-glucopyranoside)	C_21_H_32_O_11_	*D. moniliforme*	[54]
59	Dendromoniliside C (2*α*,3*α*,8*β*-trihydroxy-9*α*- (11)-epoxypicrotoxan-3(15*α*)-olide 8-*O-β-D*-glucopyranoside.)	C_21_H_32_O_10_	*D. moniliforme*	[54]
60	Dendromoniliside D (2*α*,3*α*,11,12-tetrahydroxypicrotoxan-3(15*α*)-olide 11-*O-β*-D-glucopyranoside)	C_21_H_34_O_10_	*D. moniliforme*	[54]
61	Crystallinin	C_15_H_22_O_5_	*D. crystallinum*	[55]
62	Dendronobilin J	C_15_H_22_O_6_	*D. nobile*	[48,55]
63	Dendronobilin F	C_15_H_22_O_5_	*D. nobile*	[46]
64	Dendronobilin M	C_15_H_24_O_6_	*D. nobile*	[54]
65	Dendronobilin C	C_15_H_22_O_6_	*D. nobile*	[54]
66	Dendrowillin B	C_15_H_20_O_6_	*D. williamsonii*	[25]
67	Amotin	C_15_H_20_O_6_	*D. amoenum*	[56]
68	Dendrowillin A	C_15_H_20_O_7_	*D. williamsonii*	[25]
69	Dengrasusane A	C_15_H_18_O_7_	*D. gratiosissimum*	[57]
70	Dendroterpene C	C_15_H_20_O_4_	*D. nobile*	[44]
71	Dendroterpene D	C_15_H_20_O_5_	*D. nobile*	[44]
72	Dendroterpene E	C_15_H_22_O_4_	*D. nobile*	[39]
73	Aduncin C	C_15_H_18_O_6_	*D. huoshanense*	[25]
74	Wardianumine A	C_19_H_31_NO_4_	*D. wardianum*	[29]
75	Amoenin	C_15_H_22_O_6_	*D.amoenum*	[56]

**Table 3:** Information table of allo-aromadendrane-type sesquiterpenoids isolated from *Dendrobium*.

No.	Compounds	Formula	Sources	Refs.
76	Dendronobilin H	C_15_H_26_O_3_	*D. nobile*	[46,55]
77	Dendrobiumane A	C_15_H_26_O_3_	*D. nobile*, *D. moniliforme*	[48,55]
78	Dendroside A (10*β*,12,14-trihydroxyalloaromadendrane 14-*O-β*-D- glucopyranoside)	C_21_H_36_O_8_	*D. nobile*	[51]
79	Dendroside B (10*β*,12,14-trihydroxyalloaromadendrane 12,14-di-*O-β*-D-glucopyranoside.)	C_27_H_46_O_13_	*D. nobile*	[50]
80	Dendroside D (10*β*,14-dihydroxy-alloaromadendran-12-oic acid 12,14-di-*O-β*-D-glucopyranoside)	C_27_H_44_O_14_	*D. nobile*	[47]
81	10*β*,12,14-trihydroxyalloaromadendrane	C_15_H_26_O_3_	*D. nobile*	[50]
82	Dendroside C (10*β*,13,14-trihydroxyalloaromadendrane 14-*O-β*-D-glucopyranoside.)	C_21_H_36_O_8_	*D. nobile*	[50]
83	Soltorvum F	C_17_H_28_O_4_	*D. nobile*	[55]
84	10*β*,13,14-trihydroxyalloaromadendrane	C_15_H_26_O_3_	*D. nobile*	[55]
85	Dendrofindlayanoside A	C_21_H_34_O_8_	*D. findlayanum*	[52]
86	Dendrofindlayanoside B	C_21_H_36_O_8_	*D. indlayanum*	[52]

**Table 4:** Information table of copacamphane- and cyclocopacamphane-type sesquiterpenoids isolated from *Dendrobium*.

No.	Compounds	Formula	Sources	Refs.
87	Dendronobilin A	C_15_H_24_O_3_	*D. nobile*	[55]
88	Dendronobilin K	C_15_H_24_O_4_	*D. nobile*	[46]
89	(+)-(1*R*,5*R*,6*S*,8*R*,9*R*)-8,12-dihydroxy-copacamphan-3-en-2-one	C_15_H_22_O_3_	*D. nobile*	[21]
90	Dendromoniliside A (2*α*,12-dihydroxycopacamphan-15-one 2-*O-β*-D-glucopyranoside)	C_21_H_34_O_8_	*D. moniliforme*	[54]
91	Dendronobiloside E (5*β*,12,15-trihydroxy-cyclocopacamphane 12-*O-β*-D-glucopyranoside)	C_21_H_34_O_8_	*D. nobile*	[47]
92	Dendrobane A (5*β*,8*β*,12-trihydroxy-cyclopacamphane)	C_15_H_24_O_3_	*D. nobile*	[47]
93	Dendronobilin I	C_15_H_24_O_3_	*D. nobile*	[46]
94	Dendronobilin N	C_15_H_24_O_4_	*D. nobile*	[46]
95	Dendrofindlayanobilin A	C_15_H_24_O_3_	*D. findlayanum*	[52]

**Table 5:** Information table of cadinene-type sesquiterpenoids isolated from *Dendrobium*.

No.	Compounds	Formula	Sources	Refs.
96	Dendronobiloside C (11,15-dihydroxycadinene 11,15-di-*O*-*β*-D-glucopyranoside)	C_27_H_44_O_12_	*D. nobile*	[50]
97	Dendronobiloside D (14,15-dihydroxycacinen 14,15-di-*O*-*β*-D-glucopyranosidle)	C_27_H_44_O_12_	*D. findlayanum*	[50,58]
98	Cadalene-12-*O*-*β*-D-glucopyranoside	C_21_H_28_O_6_	*D. nobile*	[59]
99	Findlayanosides A ((1*R*,6*R*,7*S*,10*S*)-muurol-4-ene-1,11,15-triol 15-*O*-b-D-glucopyranoside)	C_21_H_36_O_8_	*D. findlayanum*	[58]
100	Findlayanosides B ((1*R*,6*R*,7*S*,10*S*)-muurol-4-ene- 1,11,15-triol 11-*O*-b-D-glucopyranoside)	C_21_H_36_O_8_	*D. findlayanum*	[58]

**Table 6:** Information table of emmotin-, murolene-, axane-, and ylangene-derived-type sesquiterpenoids isolated from *Dendrobium*.

No.	Compounds	Formula	Sources	Refs.
101	Dendroside E (1*α*,13,14-trihydroxyemmotin 14-*O-β*-D-glucopyranoside)	C_21_H_36_O_8_	*D. nobile*	[47]
102	Dendronobilin G (9*β*,10*α*)-muurol-4-ene-9,10,11-triol	C_21_H_36_O_8_	*D. nobile*	[46]
103	Bullatantriol	C_15_H_28_O_3_	*D. nobile*	[60]
104	Dendrofindlayanoside C	C_22_H_36_O_8_	*D. findlayanum*	[52]

**Table 7:** The potential impact of *DSS* in diabetes mellitus.

*Dendrobium* Species or Compounds	Model	Effects and Possible Molecular Mechanisms	Refs.
DNLA	8-week-old male Kunming mice	DNLA has beneficial effects on liver glucose and lipid metabolism gene expressions, and it enhances the Nrf2-antioxidant pathway gene expressions, which could play integrated roles in regulating metabolic disorders.	[37]
*D. nobile*	HFD/STZ-induced T2DM rat	*D. nobile* might be associated with liver glycogen synthesis and gluconeogenesis, contributing to improving insulin resistance and abnormal glucose metabolism in T2DM rats.	[27]
Dendrobine	Genetic mouse model of GDM	Reducing the secretion of inflammatory cytokines by T helper 17 (Th17) cells, including interleukin-1β, interleukin-6, tumor necrosis factor-α, and interleukin-17.	[33]
*D. chrysotoxum*	STZ-induced diabetic rats	Inhibiting the expression of VEGF/VEGFR2, and some other pro-angiogenic factors such as MMP 2/9, PDGF A/B, bFGF, IGF-1.	[62]
*D. loddigesii*	3T3-L1 cells and db/db mice	Enhancing the expression levels of GLUT-4 and p-AMPK in the adipose tissue and increasing the expression levels of PPARα and p-AMPK in the liver tissue.	[48,58]

**Table 8:** The potential impact of *DSS* on brain pathology.

*Dendrobium* Species or Compounds	Model	Effects and Possible Molecular Mechanisms	Refs.
DNLA	H_2_O_2_-exposed N2A cells	Inhibit the expression of pro-inflammatory and pro-apoptotic factors by blocking JAK-STATs signaling after oxidative stress injury.	[67]
DNLA	LPS-stimulated rats	Attenuates LPS-induced hyperphosphorylation of tau protein in rat hippocampus and protects against LPS-induced apoptosis in rat brain.	[68]
DNLA	Mn-exposed PC12 cells	Alleviation of cell toxicity, apoptotic cell death with the up-regulation of Bax and down-regulation of Bcl-2, improvement of mitochondrial respiratory capacity and oxidative status, modulation of PINK1/Parkin-mediated autophagy flux mitochondria function.	[73]
*D. nobile* alkaloids	Rat primary cultured cerebral cortical neurons	Amelioration of neuronal damage in brain hypoxia-ischemia.	[69]
DNLA	Bilateral intracerebroventricular (ICV) streptozotocin (STZ) injection in rats	Ameliorate the STZ-induced hippocampal neuron injury, up-regulate the activity of GSK-3β, and inhibit the hyperphosphorylation of Tau protein in rats.	[63]
DNLA	Aβ25-35-induced PC12 cell damage or rat hippocampus primary neurons	Rescue Aβ-mediated synaptic and mitochondrial injury and inhibit amyloidogenesis in vivo and in vitro, probably through the activation of Wnt/β-catenin signaling pathway to protect synaptic integrity.	[65,70]
Alkaloid-enriched extract from *D. nobile*	Male C57/BL mice pretreated with different concentrations of DNLA underwent transient middle cerebral artery occlusion and reperfusion.	Protect against CIR damage by inhibiting pyroptosis-induced neuronal death.	[71]
DNLA	SAMP8 mice	Improve cognitive dysfunction and ameliorate neuronal injury in aged SAMP8 mice, and attenuate aberrant protein/gene expressions.	[66]
*Dendrobium* alkaloids	Elderly normal mice or Aβ25–35-induced	Increasing the expression of BDNF, GDNF, and CNTF in the hippocampus and cortex; improving Aβ-induced spatial learning and memory impairment in mice.	[74]
DNLA	Methionine-diet-fed mice	Down-regulate the expression of amyloid-precursor protein (APP), presenilin-1 (PS1), beta-secretase-1 (BACE1), DNA methyltransferase1 (DNMT1), and Aβ1–40) and Aβ1–42) proteins. Increase CPG island methylation levels of APP and BACE1 genes.	[75]
DNLA	APP/PS1 transgenic mice	DNLA was effective in improving cognitive deficits in aged SAMP8 mice, possibly via suppression of ER stress-related PERK signaling pathway; sequential inhibition of calpain 1, GSK-3β, and Cdk5 activities; and eventually reducing the hyper-phosphorylation of Tau. Sequential inhibition of calpain 1, GSK-3β, and Cdk5 activity, and eventually reducing the hyper-phosphorylation of Tau.	[76]
DNLA	Aged SAMP8 mice	DNLA and metformin treatments prevent brain atrophy and improve morphological changes in the hippocampus and cortex, as evidenced by Nissl and H&E staining for neuron damage and loss, and by SA-β-gal staining for aging cells.	[76,77]
DNLA	6-OHDA-treated mice	Prevention and treatment of Parkinson’s disease	[78]
DNLA	CUS-induced rat model of depression	DNLA attenuates HPA activation by decreasing adrenocorticotropic hormones and the expression of corticotropin-releasing hormone receptor-1, and it increases the expression of the glucocorticoid receptor in the brain.	[79]

**Table 9:** The potential impact of *DSS* on anti-tumor.

*Dendrobium* Species or Compounds	Model	Effects and Possible Molecular Mechanisms	Refs.
dendrowardin E, amotin, aduncin	Human lens epithelial cells (HLECs) induced by D-galactose	Exhibited significant effects of promoting the proliferation on human lens epithelial cells (HLECs) induced by D-galactose.	[46]
Dendrobin	A549 lung cancer cellsA549 xenograft in nude mice	Stimulation of JNK/p38 stress signaling pathways and, consequently, the induction of apoptosis involving pro-apoptotic proteins Bax and Bim.	[80]
Dendrobin	γ-Irradiation-Induced Cancer Cell Migration	Dendrobine inhibited γ-irradiation-induced migration and invasion of A549 cells by suppressing sulfatase2 (SULF2) expression, thus inhibiting IR- induced signaling.	[82]
findlayanoside C, findlayanoside D	SMMC-7721, A-549 and MCF-7 human cancer cell lines	Cytotoxic activity assays against SMMC-7721, A-549, and MCF-7 human cancer cell lines.	[17]
ethanolic extract of *D. chrysanthum*	HeLa (human cervical cancer) cells Dalton’s lymphoma (DL)-bearing mice	Mediated through p53-dependent apoptosis.	[81]

**Table 10:** The potential impact of *DSS* on inflammatory immune responses.

*Dendrobium* Species or Compounds	Model	Effects and Possible Molecular Mechanisms	Refs.
Dendroside D, dendroside F, dendroside G, dendroside A, dendronobiloside A	T and B lymphocytes	Possess anti-tumor and antimutagenic activity.	[47,51]
Dendrohercoglin A–C	RAW 264.7 cells	Cytotoxic activity assays against SMMC-7721, A-549, and MCF-7 human cancer cell lines.	[17]
Dendrobine	Patients who met the diagnostic criteria for MASLD	Downregulating inflammatory mediators like TNF, IL6, IL1B, and inhibiting AKT1 and Signal Transducer and Activator of Transcription 3 (STAT3).	[84]
*D. nobile* aqueous extract	Mice were treated with a daily alcoholic intragastric for 14 days	DNAE upregulated ELF4 expression through the RTK/ERK1/2 axis. ELF4 inhibits macrophage inflammatory responses via transactivating its upstream target genes S100A9.	[24]
